# Nitrite-Templated Synthesis of Lanthanide-Containing [2]Rotaxanes for Anion Sensing**

**DOI:** 10.1002/anie.201405131

**Published:** 2014-07-02

**Authors:** Matthew J Langton, Octavia A Blackburn, Thomas Lang, Stephen Faulkner, Paul D Beer

**Affiliations:** Chemistry Research Laboratory, Department of Chemistry, University of OxfordMansfield Road, Oxford, OX1 3TA (UK)

**Keywords:** anion, lanthanide, rotaxane, supramolecular chemistry, template synthesis

## Abstract

The first anion-templated synthesis of a lanthanide-containing interlocked molecule is demonstrated by utilizing a nitrite anion to template initial pseudorotaxane formation. Subsequent stoppering of the interpenetrated assembly allows for the preparation of a lanthanide-functionalized [2]rotaxane in high yield. Following removal of the nitrite anion template, the europium [2]rotaxane host is demonstrated to recognize and sense fluoride selectively.

Interlocked molecular architectures, such as rotaxanes and catenanes, have been the subject of intense recent research, driven by proposed utilization in molecular machines and nanotechnological applications.[1–5] Furthermore, the unique topological cavity formed between the interlocked components can be exploited for molecular recognition.[6] Whilst metal cations have been used extensively as templates for interlocked molecule synthesis,[7–12] employing anions as templates is less well developed. Discrete anion templation has, however, been demonstrated as an efficient route to interlocked molecule formation,[6] with the resulting host structures displaying enhanced selectivity for binding the templating anion. Although a range of templating anions have been utilized for rotaxane and catenane synthesis, including halides,[13,14] sulfate,[15,16] and most recently nitrate,[17] the use of the nitrite anion as a template in synthesis is, to the best of our knowledge, unprecedented. Incorporating redox- or photo-active reporter groups within interlocked host architectures enables the development of sensors which exhibit selective electrochemical or optical responses upon guest binding.[18] However, while phosphorescent transition metal complexes and organic fluorophores have been extensively utilized in such optical anion sensors, the integration of lanthanide reporter groups within the binding cavity of such interlocked hosts has not been achieved,[19] despite the usefulness of emissive lanthanide complexes in imaging, sensing, and assay.[20,21]

We describe herein the first anion-templated synthesis of a lanthanide-containing interlocked molecule, by the unprecedented use of a nitrite anion template. The [2]rotaxane host system, isolated in high yields, is capable of sensing fluoride anions selectively by means of the luminescent europium center integrated within the rotaxane’s macrocycle component.

The nitrite anion templation synthetic strategy used to prepare the target lanthanide-[2]rotaxane is shown in Scheme 1. The bidentate nitrite anion templates the formation of an initial pseudorotaxane, by simultaneously coordinating to the lanthanide cation bound within a kinetically stable DOTA-derived (DOTA=1,4,7,10-tetraazacyclododecane-1,4,7,10-tetraacetic acid) macrocycle, and to the hydrogen bonding bis-amide pyridinium motif of the axle precursor. Stoppering of this interpenetrated assembly affords the [2]rotaxane, which after removal of the templating nitrite anion, reveals an anion-binding cavity formed between the interlocked lanthanide macrocycle and the axle bis-amide pyridinium motif.

**Scheme 1:**
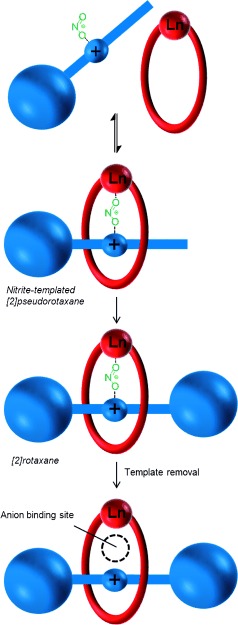
Schematic representation of nitrite templation of a lanthanide-containing [2] rotaxane for anion recognition and sensing, by mono-stoppering of a [2]pseudorotaxane.

Macrocycle **1⋅OTf**[11] and the chloride salt of azide-functionalized bis-amide pyridinium axle precursor **2^+^** [22] were prepared as previously described. **2⋅NO_2_** was prepared by anion exchange from the chloride salt using a nitrite-loaded Amberlite anion-exchange resin. The synthesis of the target lanthanide rotaxanes was achieved using a copper(I)-catalyzed cycloaddition azide–alkyne (CuAAC) click stoppering reaction as shown in Scheme 2.

**Scheme 2:**
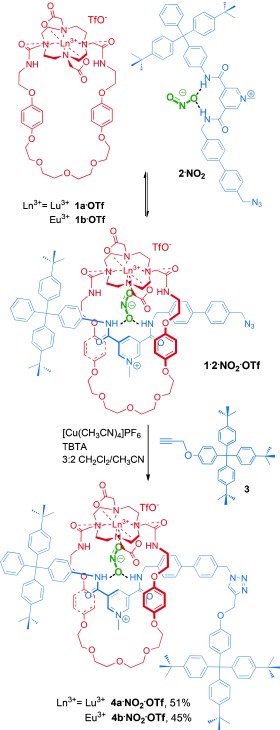
Nitrite-templated synthesis of lanthanide [2]rotaxanes 4 a⋅NO_2_⋅OTf and 4 b⋅NO_2_⋅OTf. TBTA=tris-[(1-benzyl-1H-1,2,3-triazol-4-yl)methyl]amine.

A solution of 1 equivalent of **2⋅NO_2_** with 1.5 equivalents of the appropriate macrocyclic lanthanide complex (**1 a⋅OTf** and **1 b⋅OTf**) in dry 3:2 CH_2_Cl_2_/CH_3_CN was stirred in the presence of a catalytic amount of [Cu(CH_3_CN)_4_]PF_6_ and 1 equivalent of stopper alkyne **3** at room temperature for 48 h. Following purification by size exclusion chromatography, to remove the non-interlocked macrocycle and axle byproducts, rotaxanes **4 a⋅NO_2_⋅OTf** and **4 b⋅NO_2_⋅OTf** were isolated in impressive yields of 51 % and 45 %, respectively, and characterized by NMR spectroscopy, mass spectrometry, and HPLC (see Supporting Information (SI) and Figure 1). The ^1^H NMR spectrum of **4 a⋅NO_2_⋅OTf** was broad, even in [D_6_]DMSO at 353 K (see SI). However, the upfield shift and splitting of the hydroquinone protons of the macrocycle is clearly observed and is diagnostic of aromatic donor–acceptor interactions between the electron-rich hydroquinone groups of the macrocycle and the electron-deficient pyridinium axle motif, consistent with the interlocked nature of the rotaxane. HPLC analysis was used to establish the purity of rotaxanes **4 a⋅NO_2_⋅OTf** and **4 b⋅NO_2_⋅OTf**, which exhibited marked differences in retention times to the non-interlocked axle and macrocycle components, providing further evidence as to the interlocked nature of the rotaxane species (see SI). In addition, mass spectrometry provided further structural characterization of the interlocked rotaxane systems (see Figure 1 and SI for further details).

**Figure 1:**
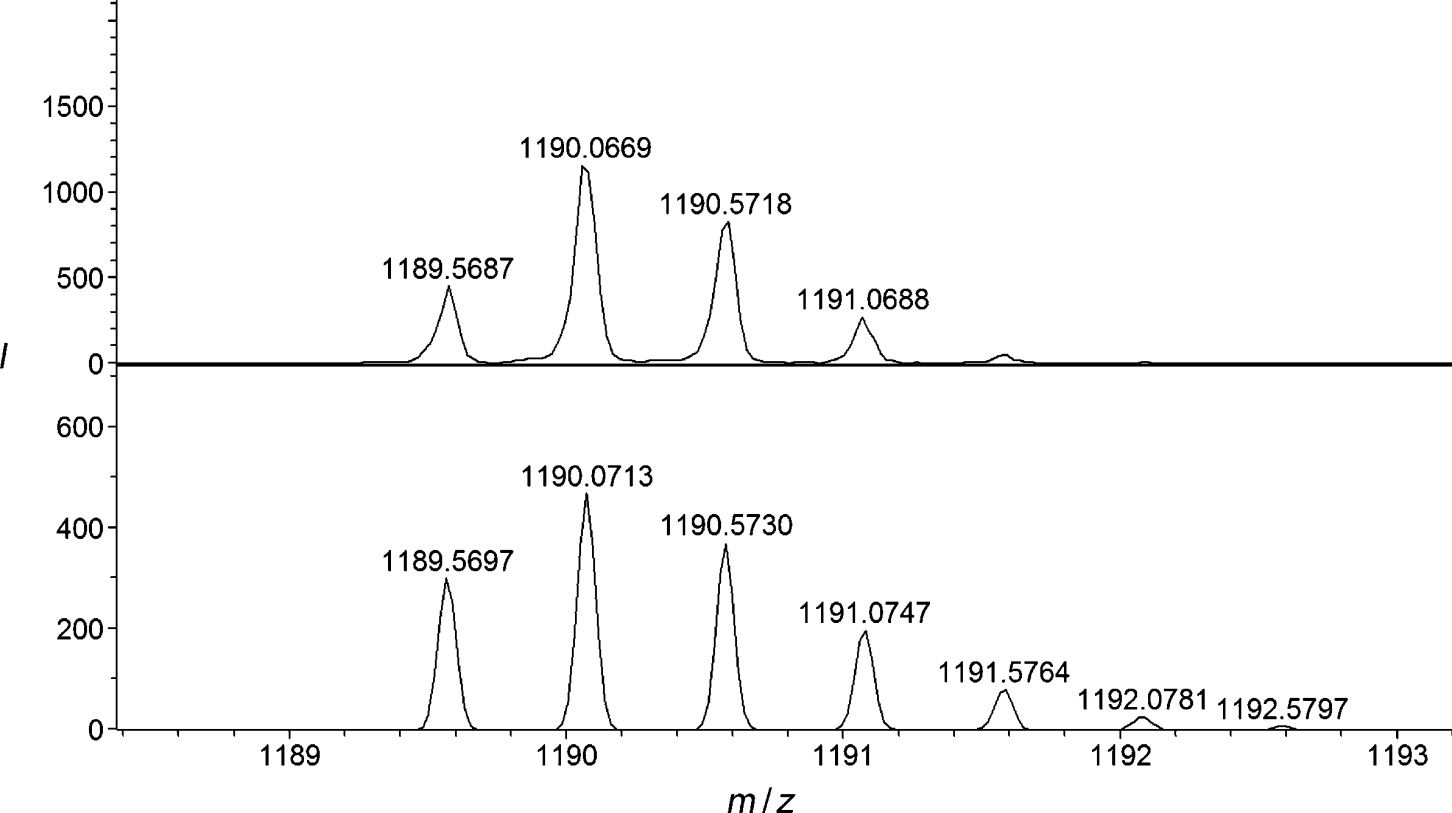
High resolution mass spectrum of [2]rotaxane 4 a⋅NO_2_⋅OTf (top) with theoretical isotope model for [*M*−TfO^−^−NO_2_^−^]^2+^ (bottom).

Importantly, the rotaxanation reaction was also attempted using the non-coordinating hexafluorophosphate salt of axle precursor **2⋅PF_6_**, however, no rotaxane was detected in the reaction mixture, highlighting the crucial role of the nitrite anion in templating rotaxane formation. The rotaxane synthesis was also attempted using Cl^−^ and F^−^ as the templating anion: when the reaction was conducted with **2⋅Cl** only a trace amount of rotaxane was observed by mass spectrometry and could not be isolated, whilst with F^−^, added as the tetrabutylammonium (TBA) salt to **2⋅PF_6_**, no evidence of rotaxane formation was detected.

In order to investigate the anion binding properties of rotaxane **4 b^2+^**, it was necessary to remove the nitrite anion template by exchanging to the weakly coordinating triflate salt. Luminescence anion binding titrations were conducted with **1 b⋅OTf** and **4 b⋅(OTf)_2_** in 99:1 acetone/water solution, by adding aliquots of anions as their TBA salts and monitoring changes in emission intensity from the europium center.[23] Association constants were determined by fitting intensity changes to a 1:1 stoichiometric binding model using the Dynafit program;[24] the values obtained are given in Table 1. Representative data are shown in Figure 2; all other spectra and binding isotherms are provided in the Supporting Information. Addition of fluoride to rotaxane **4 b⋅(OTf)_2_** resulted in a drastic decrease in emission intensity from the europium center, down to 10 % of its original value at the end of the titration (Figure 2). In contrast, addition of acetate and nitrite led to more modest quenching, whilst no response was observed upon addition of chloride. Likewise, addition of anions to macrocycle **1 b⋅OTf** resulted in similar quenching of the europium emission, but to a lesser extent than in rotaxane **4 b⋅(OTf)_2_**. The effect of fluoride on the europium emission of **1 b⋅OTf** and **4 b⋅(OTf)_2_** is also evident by inspection of the luminescence lifetimes (Table 2). The significant lengthening of the lifetimes of **1 b⋅OTf** and **4 b⋅(OTf)_2_** upon addition of fluoride is indicative of displacement of water from the inner coordination sphere of the metal, and is consistent with binding of fluoride at the lanthanide center. Further evidence for this can be gleaned from the marked change in the intensity of the hypersensitive Δ*J*=2 bands relative to the other bands in the spectra (see SI), which signifies a change in environment about the metal. By comparison, nitrite has apparently no effect on the luminescence lifetimes of **1 b⋅OTf** or **4 b⋅(OTf)_2_**, and there is no significant change in the ratios of Δ*J*=2 vs. Δ*J*=1 or Δ*J*=4 over the course of the titrations. The quenching of europium emission in this case can be assigned to an attractive association of the anion with the organic moieties of the interlocked architecture, which leads to less efficient energy transfer from the chromophore to lanthanide, while the latter remains hydrated. Acetate presents an intermediate case in which the lifetimes are increased, but to a lesser degree than with fluoride. This infers a combination of effects resulting from anion binding at the metal and interaction with the organic host framework.

**Figure 2:**
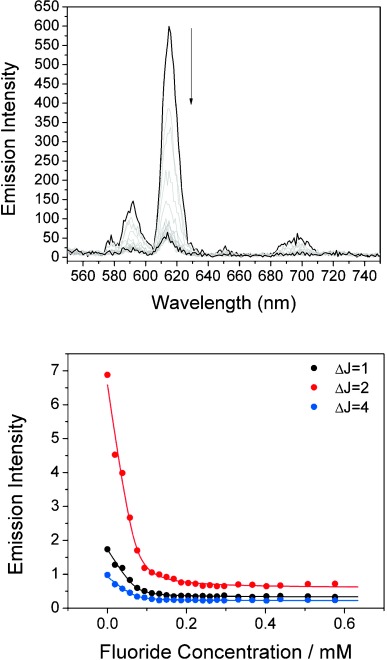
Titration of a 5×10^−5^ m solution of rotaxane 4 b⋅(OTf)_2_ with tetrabutylammonium fluoride (TBA^.^F) in 99:1 acetone/H_2_O. Top: Changes in the emission spectra upon 360 nm excitation. Bottom: Binding isotherms for europium emission bands and the best global fit obtained.

**Table 1:** Association constants *K*_a_ [m^−1^] for 1 b⋅OTf and 4 b⋅(OTf)_2_ in 99:1 acetone/water determined from luminescence data.^[a]^

Anion^[b]^	Rotaxane4 b⋅(OTf)_2_	Macrocycle1 b⋅OTf
F^−^	2.42×10^5^ [1.08–8.15×10^5^]	9.69×10^4^ [7.75–12.5×10^4^]
AcO^−^	2.42×10^4^ [1.94–3.08×10^4^]	1.55×10^3^ [1.11–2.13×10^3^]
NO_2_^−^	4.33×10^3^ [3.30–5.62×10^3^]	1.10×10^4^ [0.67–1.76×10^4^]
Cl^−^	–^[c]^	–^[c]^

[a] The values in square brackets show 95 % confidence intervals. [b] Anions added as TBA salts. [c] Addition of Cl^−^ caused no change in luminescence intensity.

**Table 2:** Eu^3+^-based luminescence lifetimes [ms] at 616 nm of 1 b⋅OTf and 4 b⋅(OTf)_2_ without added anions, and at the end of the titrations.

Anion^[a]^	Rotaxane4 b⋅(OTf)_2_	Macrocycle1 b⋅OTf
no anion	0.69, 0.16	0.51
F^−^	1.12, 0.24	1.27
AcO^−^	0.87, 0.16	0.91
NO_2_^−^	0.63, 0.11	0.46

[a] Anions added as TBA salts.

The anion association constants obtained (Table 1) suggest superior binding of fluoride to both macrocycle **1 b⋅OTf** and rotaxane **4 b⋅(OTf)_2_** compared with nitrite and acetate. Bearing in mind the above discussion, this implies that anion association at the europium metal center is most effective. For fluoride and acetate, the rotaxane system **4 b⋅(OTf)_2_** displays stronger binding than the macrocycle **1 b⋅OTf**. This indicates that formation of the interlocked structure with additional hydrogen bond donor groups augments anion binding at the lanthanide center. In the case of nitrite, the anion is unable to displace the coordinated water molecule at the lanthanide center, and the additional hydrogen bond donors confer no enhancement of nitrite association in the interlocked structure. Indeed, binding is marginally weaker than in the macrocycle alone, presumably due to steric constraints.

With the aim of developing functional anion sensory assemblies that exploit both the advantageous properties of lanthanide luminescence and the unique recognition properties of mechanically bonded architectures, the first anion-templated synthesis of a lanthanide-containing interlocked structure has been demonstrated. The bidentate coordination behaviour of a nitrite anion is used to template the formation of a pseudorotaxane assembly, by simultaneous coordination to a lanthanide cation incorporated within a macrocycle and a hydrogen-bond donor threading component. The novel [2]rotaxanes are prepared in high yield by stoppering of these interpenetrated assemblies. Crucially, the nitrite anion template is essential for rotaxane formation, with no product formed in its absence. It is noteworthy that, to the best of our knowledge, nitrite has not previously been used in template-directed synthesis. Luminescence anion binding investigations with the europium containing [2]rotaxane host reveals its selectivity for fluoride over acetate, nitrite and chloride anions.
