# Supplementary material for: Nitrite-Templated Synthesis of Lanthanide-Containing [2]Rotaxanes for Anion Sensing
Source: Angew Chem Int Ed Engl. 2014 Jul 2;53(43):11463–6. doi: 10.1002/anie.201405131 (PMC4497609; doi:10.1002/anie.201405131)
Supplement: Supplementary file 1 [file anie0053-11463-sd1.pdf]

Angewandte Chemie
Eine Zeitschrift der Gesellschaft Deutscher Chemiker

Supporting Information

© Wiley-VCH 2014

69451 Weinheim, Germany

Nitrite-Templated Synthesis of Lanthanide-Containing [2]Rotaxanes for Anion Sensing**

Matthew J. Langton, Octavia A. Blackburn, Thomas Lang, Stephen Faulkner, and Paul D. Beer**

anie_201405131_sm_miscellaneous_information.pdf

TABLE OF CONTENTS

Part I.

**A**	General considerations	S2
**B**	Synthesis and structural characterisation of rotaxanes **4a**·**NO$_2$**·**OTf** and **4b**·**NO$_2$**·**OTf**	S3

Part II. Luminescence studies S6

Part I: Experimental Procedures.

A. General Considerations

All solvents and reagents were purchased from commercial suppliers and used as received unless otherwise stated. Dry solvents were obtained by purging with nitrogen and then passing through an MBraun MPSP-800 column. H_2O was de-ionised and micro filtered using a Milli-Q ® Millipore machine. TBA salts were stored in a vacuum desiccator containing phosphorus pentoxide prior to use. Triethylamine was distilled from and stored over potassium hydroxide. Size exclusion chromatography was carried out using Biobeads SX-1, with $CHCl_3$ as the eluent. NMR spectra were recorded on a Bruker AVII 500 (with cryoprobe) and Bruker AVIII 500 spectrometers. Mass spectra were carried out on a Waters Micromass LCT and Bruker microTOF spectrometers. HPLC analysis was carried out using a Gilson 322-H2 HPLC instrument, with a UV-vis detector set to 254 nm, and a reverse phase cyano analytical column (Discovery® Cyano HPLC Column: 5 µm particle size, L × I.D: 25 cm × 4.6 mm). Analytical chromatography was conducted using MeCN/H_2O +0.1% Trifluoroacetic acid, using a

a gradient changing from 5:95 to 95:5 over 20 minutes, at a flow rate = 1 mL/min.

B. Synthesis and structural characterisation of rotaxanes $4a^{2+}$ and $4b^{2+}$

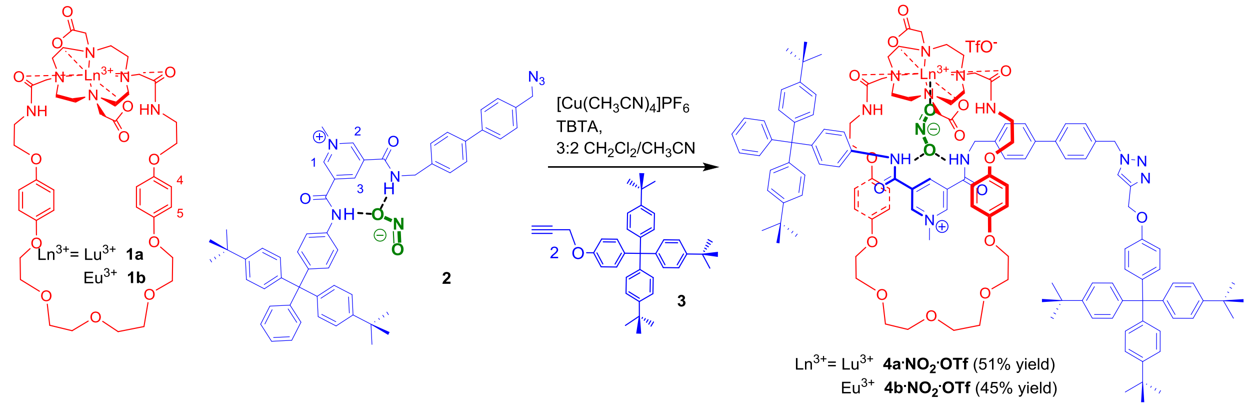

General procedure for the synthesis of rotaxanes **4a·NO$_2$·OTf** and **4b·NO$_2$·OTf**: To a solution of the corresponding macrocyclic lanthanide complex **1a·OTf** or **1b·OTf** (1.5 equiv, 0.024 M) in a 3:2 mixture of anhydrous acetonitrile/dichloromethane, compound **2·NO$_2$** (1.0 equiv) was added (prior converted from the synthesised iodide salt using an Amberlite® (nitrite) anion exchange column). The reaction mixture was stirred for 30 min and then stopper alkyne **3** (1 equiv), Cu[$(CH_3CN)_4PF_6$] (0.5 equiv), TBTA (0.5 equiv), and diisopropylethylamine (2 equiv) were added. The reaction was stirred for 48hrs at RT under N_2. After this time the solvent was evaporated under reduced pressure, the crude was dissolved in $CHCl_3$, and washed with aqueous EDTA (2 x 5 ml) and then with water (5 ml). The organic phase was dried over $MgSO_4$ and solvent removed in *vacuo*. The resulting solid was purified by using size exclusion chromatography with chloroform to give the rotaxane product: (**4a·NO$_2$·OTf** in 51% yield, **4b·NO$_2$·OTf** in 45% yield). Compound **4b·NO$_2$·OTf** was exchanged to the triflate salt by passing down a triflate-loaded Amberlite® column three times as a solution in 9:1 acetone/water to afford **4b·(OTf)$_2$** in quantitative yield. ^{1}H NMR of **4a·NO$_2$·OTf** (500 MHz, d_6-DMSO, 353K) δ: 11.76, 10.75, 10.49, 9.77, 9.58, 9.53, 9.45, 9.34, 9.21, 8.84, 8.68, 8.22, 7.67, 7.51, 7.40, 7.31, 7.21, 7.11, 7.06, 7.01, 6.91, 6.54, 6.46, 6.41, 5.62, 5.10, 4.50, 4.43, 4.19, 4.04, 3.81, 3.72, 3.59, 2.65, 2.60, 2.37, 1.32. **MS (ESI)** Compound **4a·NO$_2$·OTf :** *m/z:* 1189.5687, $[M\text{-}NO_2^-\text{-}CF_3SO_3^-]^{2+}$requires 1189.5697. Compound **4b·NO$_2$·OTf :** *m/z:* 2355.1137, $[M\text{-}NO_2^-\text{-}CF_3SO_3\text{-}H]^+$ requires 2355.1145. The synthesis of the rotaxanes were conducted on the following scales: Rotaxane **4a·NO$_2$·OTf**: macrocycle **1a·OTf** (24 mg, 0.024 mmol) and **2·NO$_2$** (15 mg, 0.016 mmol); rotaxane **4b·NO$_2$·OTf**: macrocycle **1b·OTf** (17 mg, 0.017 mmol) and **2·NO$_2$** (10 mg, 0.011 mmol).

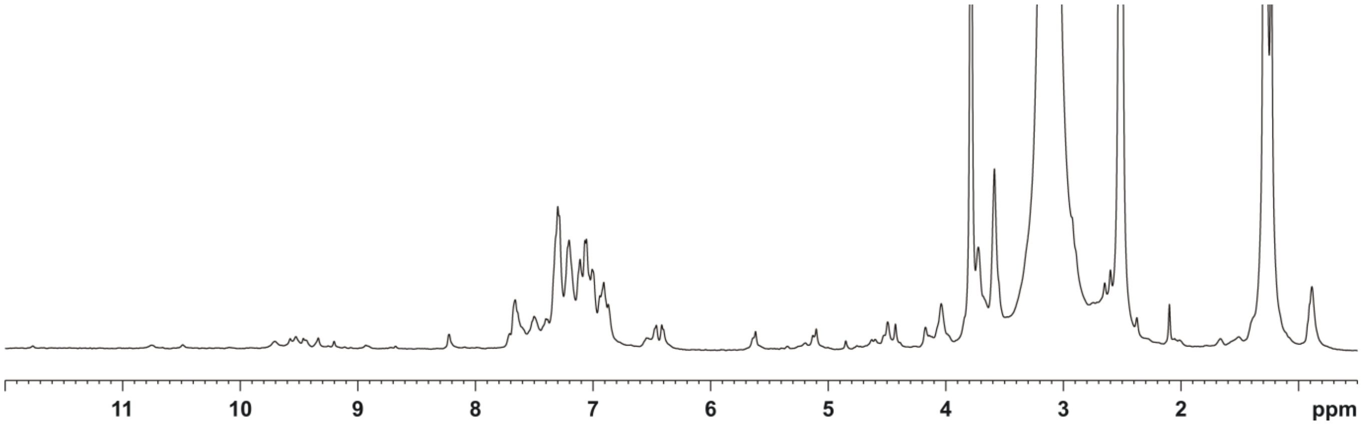

Figure SI 1. ^{1}H NMR spectrum of rotaxane **4b·NO$_2$·OTf** in d_6-DMSO (353K, 500 MHz)

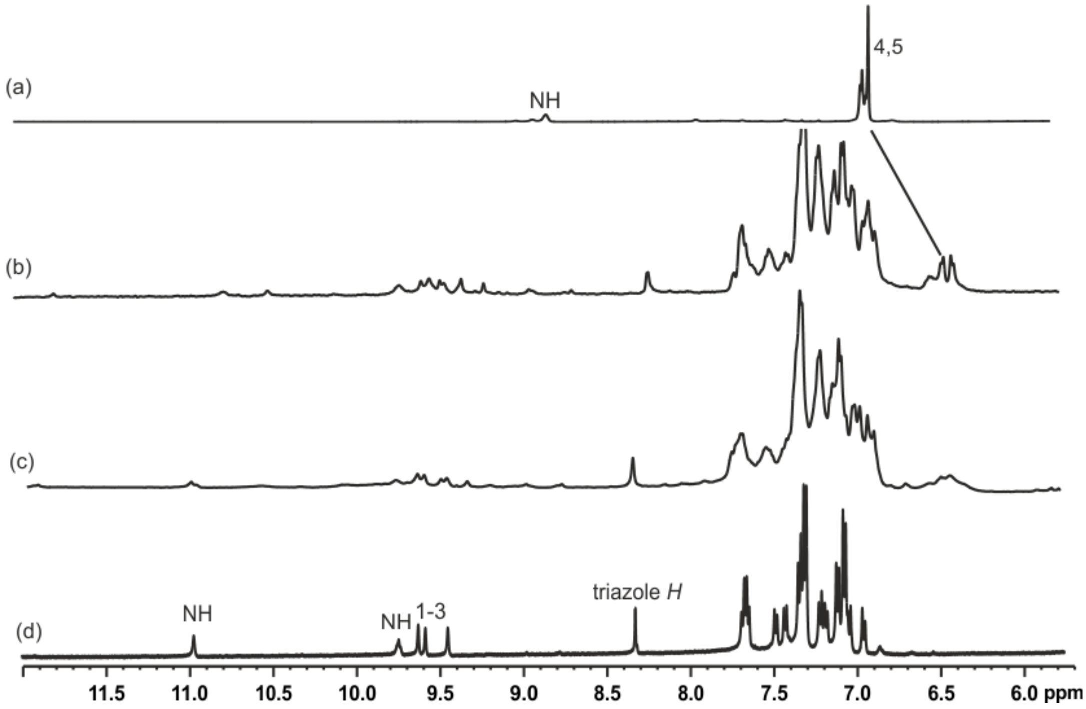

Figure SI 2. Comparison of partial ^{1}H NMR spectra in d_6-DMSO (500 MHz) of (a) Macrocycle **1a·OTf** (298K), (b) rotaxane **4b·NO$_2$·OTf** at 353K, (c) rotaxane **4b·NO$_2$·OTf** at 298K, and (d) axle

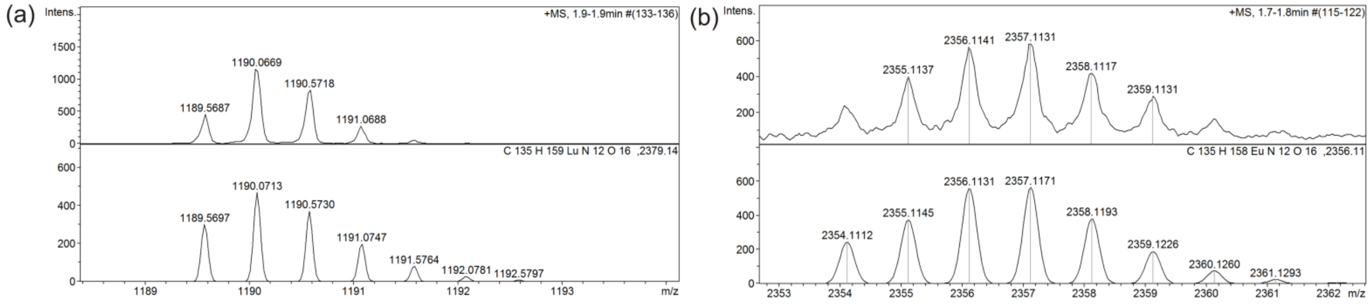

Figure SI 3. High resolution electrospray ionization mass spectrum of lanthanide rotaxanes (a) **4a·NO$_2$·OTf** and (b) **4b·NO$_2$·OTf**.

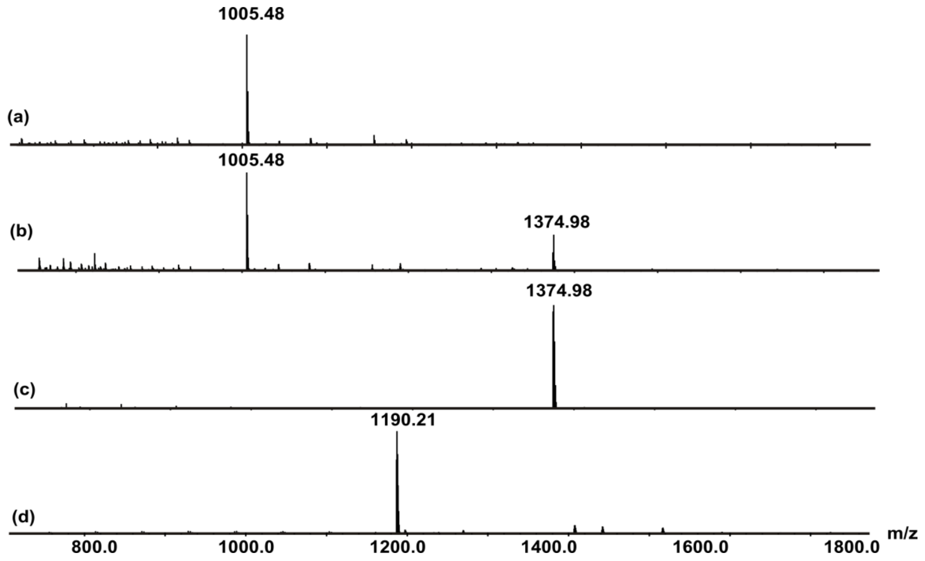

Figure SI 4. Comparison of low resolution electrospray ionization mass spectra, scanning between m/z = 750 to m/z = 2500 for samples of: (a) Macrocycle **1a·OTf**, (b) 1:1 mixture of macrcocyle **1a·OTf** and the non-interlocked axle byproduct, (c) non-interlocked axle byproduct and (d) Rotaxane **4a·NO$_2$·OTf**

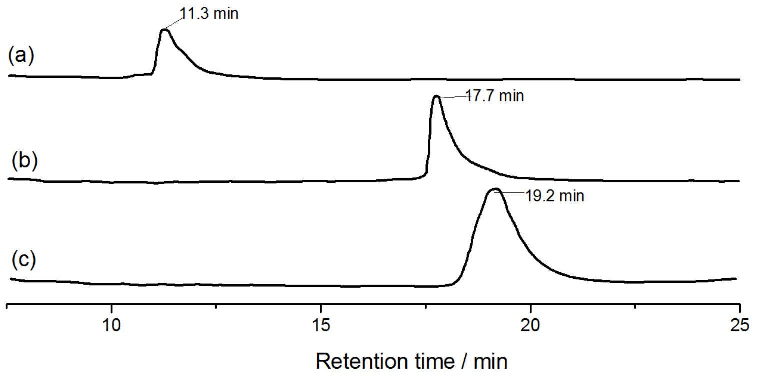

Figure SI 5. Comparison of HPLC chromatograms of (a) Macrocycle **1a·OTf**, (b) Rotaxane **4a·NO$_2$·OTf** and (c) non-interlocked axle byproduct. Retention times indicated.

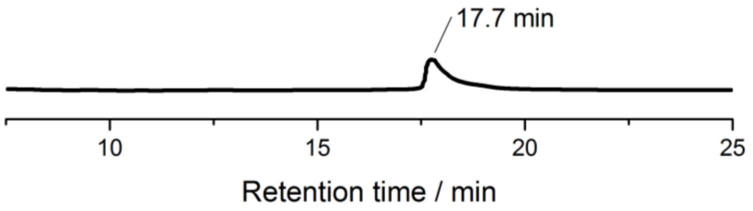

Figure SI 6. HPLC chromatogram of Rotaxane **4b·NO$_2$·OTf**

Part II: Luminescence studies

General Procedures

All luminescence spectra and lifetimes were obtained using a Horiba Fluorolog 3. Spectra were collected in phosphoresecnce mode exciting at 360 nm using a flash delay of 0.05 ms. Titrations were carried out by starting with 1.5 mL of a 5×10^{-5} M solution of the rotaxane or macrocycle and adding aliquots of a solution containing the same concentration of the rotaxane/macrocycle together with a known concentration of the anion under study. The change of intensity of each of the bands were determined by summing the intensity values across the band. Non-linear Stern-Volmer plots indicated that the decreasing luminescence is not due to collisional quenching. Lifetimes are quoted with an error of ±10%.

Figures SI 7a-f. Spectra, binding isotherms with fits and ratios of the ΔJ=2 band vs. ΔJ=1 and ΔJ=4.

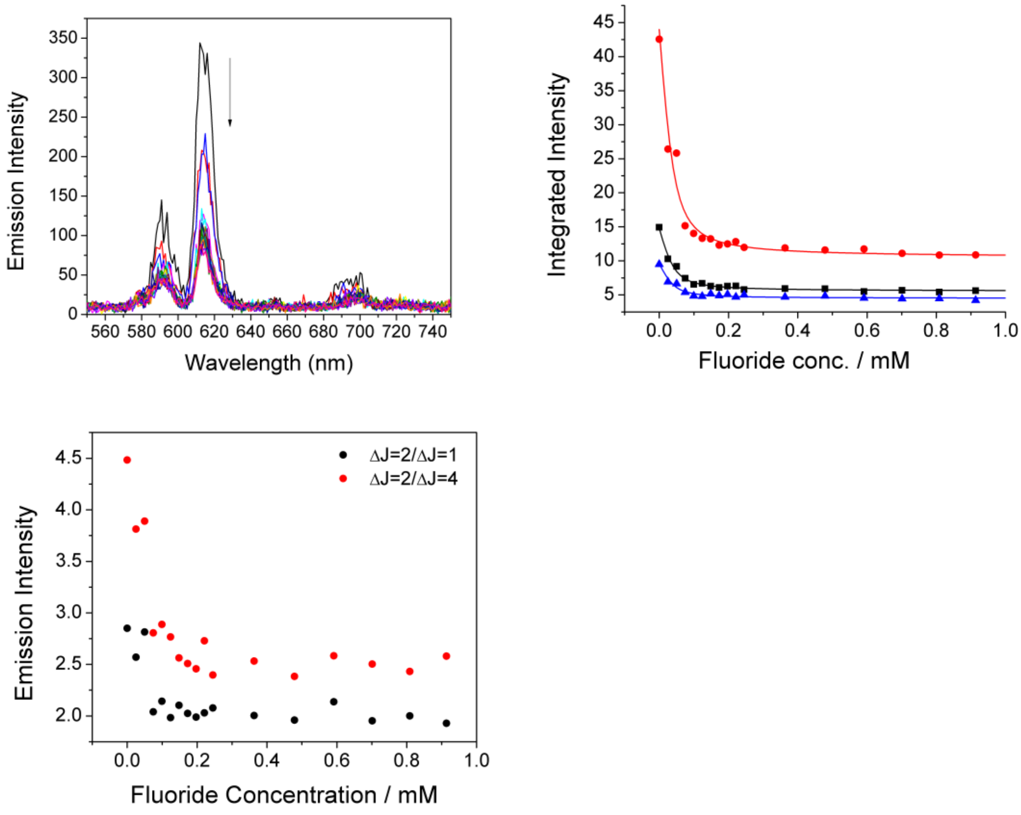

Figure SI 7a. Macrocycle + Fluoride

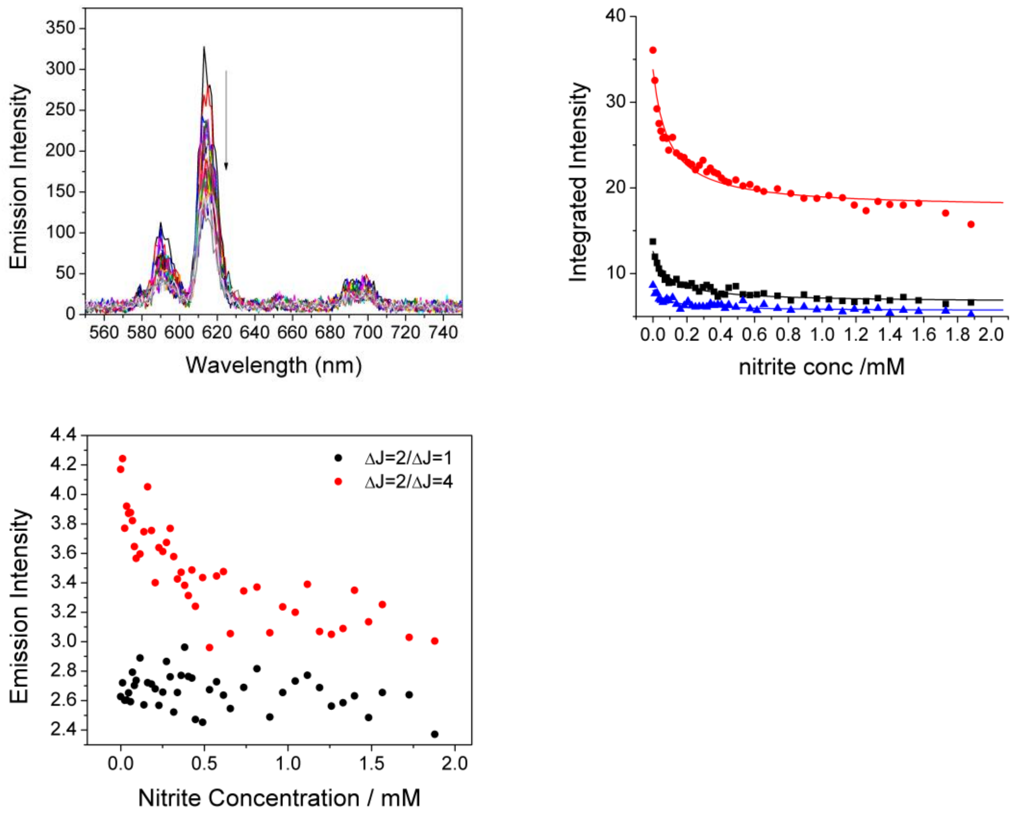

Figure SI 7b. Macrocycle + Nitrite

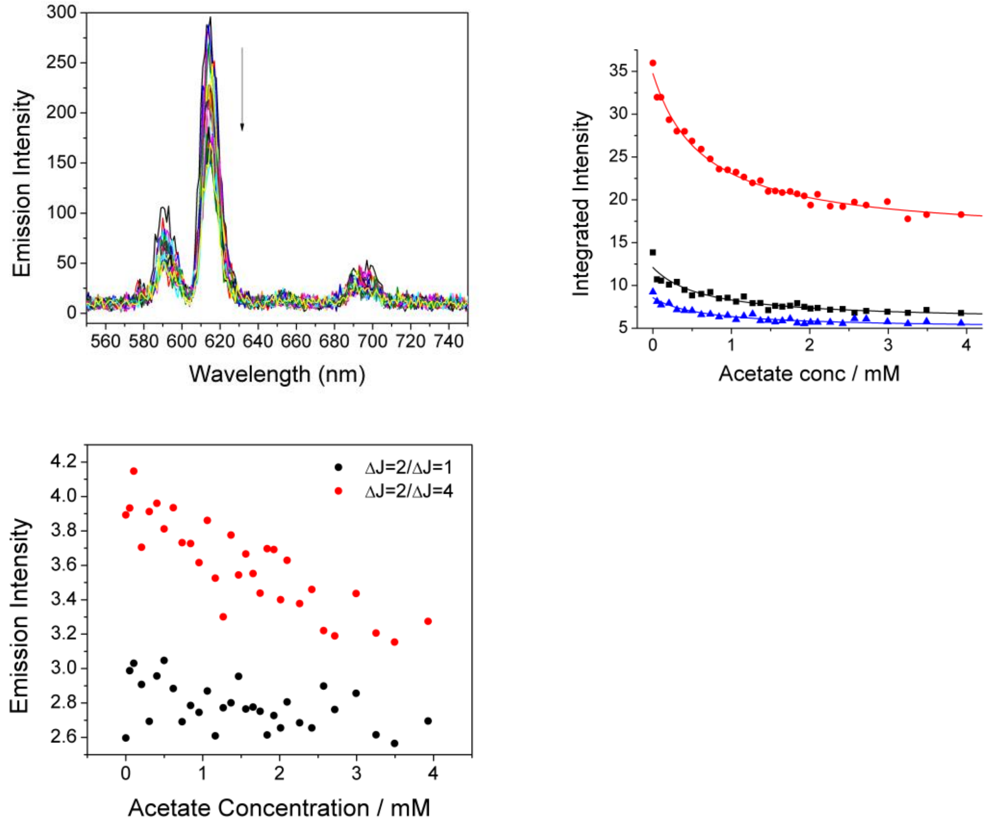

Figure SI 7c. Macrocycle + Acetate

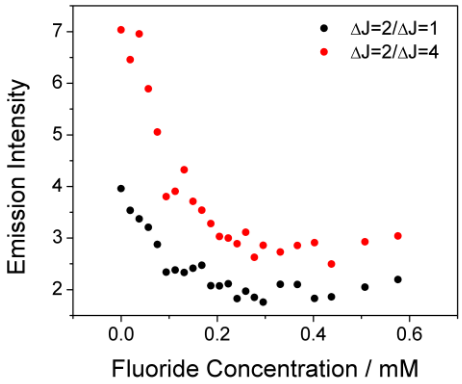

Figure SI 7d. Rotaxane + Fluoride (for binding curves and spectra see main text)

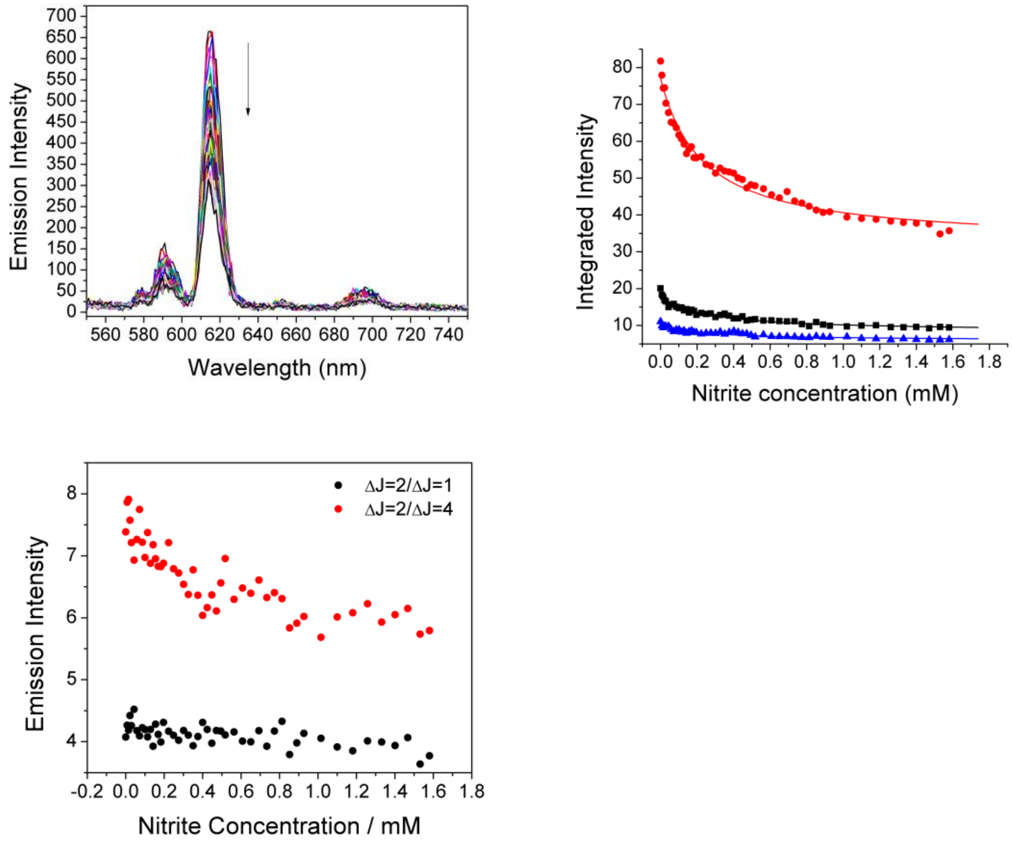

Figure SI 7e. Rotaxane + Nitrite

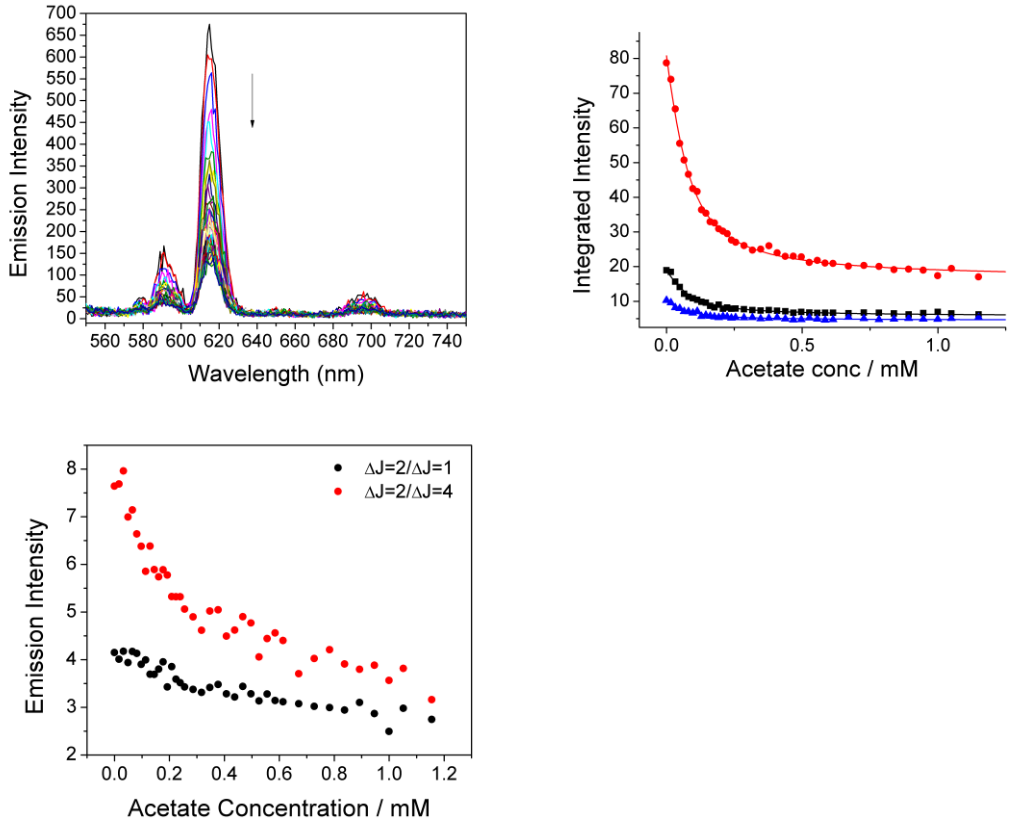

Figure SI 7f. Rotaxane + Acetate
